# DAGLα Inhibition as a Non-invasive and Translational Model of Episodic Headache

**DOI:** 10.3389/fphar.2020.615028

**Published:** 2021-01-12

**Authors:** Aidan Levine, Erika Liktor-Busa, Kelly L. Karlage, Luigi Giancotti, Daniela Salvemini, Todd W. Vanderah, Tally M. Largent-Milnes

**Affiliations:** ^1^ Department of Pharmacology, University of Arizona, Tucson, AZ, United States; ^2^ Department of Pharmacology and Physiology, Saint Louis University, St. Louis, MO, United States; ^3^ Henry and Amelia Nasrallah Center for Neuroscience, Saint Louis University School of Medicine, St. Louis, MO, United States

**Keywords:** headache, migraine, translational models, endocannabinoid system, DAGL, clinical endocannabinoid deficiency

## Abstract

Recent findings suggested that Clinical Endocannabinoid Deficiency underlies the pathophysiology of pain disorders, including migraine and headache. In models of medication overuse headache induced by sustained administration of sumatriptan or morphine, 2-AG levels were selectively depleted in the periaqueductal gray (PAG) and anandamide (AEA) increased in the cortex suggesting distinct regulation of the endocannabinoid system during headache pain. These results led to the hypothesis that blockade of DAGL, to reduce 2-AG levels would induce headache-like behaviors as a new, translationally relevant model of episodic headache. Our study investigated whether non-selective and selective blockade of DAGL, the main biosynthetic enzyme for 2-AG, induced periorbital and hind-paw allodynia, photophobia, anxiety-like behaviors, responsivity to abortive anti-migraine agents, and 2-AG/AEA levels. Injection of non-selective DAGL (DH376, 10 mg/kg, IP) and selective DAGLα (LEI106, 20 mg/kg, IP) inhibitors, but not DAGLβ agents, induced facial sensitivity in 100% and ∼60% of female and male rats, respectively, without induction of peripheral sensitivity. Notably, male rats showed significantly less sensitivity than female rats after DAGLα inhibition, suggesting sexual dimorphism in this mechanism. Importantly, LEI106 induced periorbital allodynia was attenuated by administration of the clinically available abortive antimigraine agents, sumatriptan and olcegepant. Selective DAGLα inhibition induced significant photophobia as measured by the light-dark box, without anxiety like behaviors or changes in voluntary movement. Analysis of AEA and 2-AG levels at the time of peak pain sensitivity revealed reductions in 2-AG in the visual cortex and periaqueductal gray (PAG), without altering anandamide or significantly increasing diacylglycerol levels. These results provide foundational evidence for DAGL-2AG in the induction of headache-like pain and photophobia without extracephalic allodynia, thus modeling the clinical episodic migraine. Mechanistically, behavioral measures of headache sensitivity after DAGL inhibition suggests that reduced 2-AG signaling in the cortex and PAG, but not the trigeminal nucleus caudalis or trigeminal ganglia, drives headache initiation. Therefore, episodic DAGL inhibition, which reduces the time, cost, and invasiveness of currently accepted models of headache, may fill the need for episodic migraine/headache models mirroring clinical presentation. Moreover, use of this approach may provide an avenue to study the transition from episodic to chronic headache.

## Introduction

Headache disorders, including migraine and tension-type headache (TTH), affect nearly 46% of the global population (Stovner et al., 2007; IHS, 2018). While the exact pathophysiology of these disorders has yet to be elucidated, recent findings have suggested that the endocannabinoid system (eCB) may play an important role. Low levels of the two main eCB signaling molecules, 2-arachidonoylglycerol (2-AG) and anandamide (AEA), have been correlated with pain disorders such as migraine and fibromyalgia providing clinical evidence for the theory of Endocannabinoid Deficiency (ED) (Russo, 2016).

The eCB system comprises the small lipid signalers, 2-AG and AEA, which are primarily synthesized from the cellular lipid membrane via the enzymes diacylglycerol lipase (DAGL) and N-acyl-phosphatidylethanolamine hydrolyzing phospholipase D (NAPE-PLD), respectively (Ahn et al., 2008; Fezza et al., 2016). Two types of DAGLs, DAGLα and DAGLβ are described as sn-1 speciﬁc DAGLs that synthesize 2-AG in cells (Reisenberg et al., 2012). DAGLα was originally considered as primarily central in location, with DAGLβ as a peripheral DAGL. However, knock-out studies revealed that certain DAGLα knockout mice showed 80% reduction in 2-AG levels in the brain and spinal cord, and an approximately 60% reduction in the liver. A second line of DAGLα knockout mice revealed that cerebellum, hippocampus and striatum showed the lowest level of 2-AG; no appreciable changes in central 2-AG were observed in the DAGLβ knockout mice (Gao et al., 2010; Tanimura et al., 2010). These experiments concluded that DAGLα appears to be much more important in synapse-rich regions with limited contributions from DAGLβ. After synthesis, 2-AG and AEA act on the cannabinoid receptors CB1 and CB2, with CB1 representing the primary cannabinoid receptors in the central nervous system (CNS) (Ahn et al., 2008); CB2 receptors are reported in glial cells and in some neurons (Onaivi et al., 2006; Turcotte et al., 2016). Upon activation, the CB receptors canonically function as a presynaptic G_i/o_ class of G protein coupled receptor to decrease neurotransmitter release (Mackie, 2006).

Migraine has been described as a state of CNS hyperexcitability (Aurora and Wilkinson, 2007); thus, it follows that decreased activation of presynaptic inhibitory mechanisms may lead to increased neuronal excitability, resulting in migraine induction. Several studies showed that the endocannabinoid system is centrally and peripherally engaged during pain signaling (Kaur et al., 2016; Woodhams et al., 2017). Some experimental evidence suggested eCB-dependent mechanisms underly the cause of migraine/headache; pharmacological manipulation of eCB tone by inhibiting its hydrolyzing enzymes, or directly targeting eCB receptors, seemed to represent a promising mechanistic tool and therapeutic approach to reduce migraine-like pain (Greco et al., 2018a; Tassorelli et al., 2019). Despite the mounting evidence, the real pathological connection between the eCB system and migraine with special emphasis on 2-AG signaling is not fully elucidated.

Based on the theory of CED and the therapeutic potential of elevated eCB tone in headache models (Lau et al., 2014; Nozaki et al., 2015; Zubrzycki et al., 2017; Greco et al., 2018b; Greco et al., 2020), we hypothesized that decreased levels of endocannabinoids, mainly 2-AG via exogenous inhibition of DAGL can induce migraine-like pain, and it can be utilized as a viable preclinical model for investigating headache-like pain. It can also be a useful tool to clarify the pathophysiological role of the eCB system in migraine and can support the validation of new therapeutic targets. The current study presents an original protocol for inducing headache-like pain by depletion of endocannabinoid tone, using non-selective DAGL and selective DAGLα inhibitors. Results indicate induction of cephalic hypersensitivity in the absence of extracephalic hypersensitivity following inhibition of DAGLα, but not DAGLβ, that are coupled to reductions in central, but not peripheral 2-AG levels. Allodynia induced by DAGL inhibition is responsive to two clinically relevant abortive anti-migraine agents, sumatriptan and olcegepant. DAGLα inhibition induced both head-tucking and photophobia, indicating both distress and visual sensitivity, characteristics of clinical headache. Thus, DAGL inhibition represents a new strategy to study headache that recapitulates the clinical features of headache and/or migraine as a reverse translational strategy to model CED.

## Materials and Methods

### Drugs

Ketamine, xylazine, and isoflurane were purchased from VetOne (IL, United Stated). DH376 was a generous gift from Prof. Mario van der Stelt, Leiden University. LEI106 was purchased from Cayman Chemicals (Ann Arbor, MI). KT109, olcegepant, sumatriptan, and morphine were purchased from Sigma-Aldrich (St. Louis, MO). LEI106, KT109, and olcegepant were dissolved in DMSO-Tween80-saline 0.9% (1:1:8, v/v/v). LEI106 was injected intraperitoneally at doses of 10, 20, and 40 mg/kg. KT109 was interperitoneally administered at 5, 10, 20 mg/kg doses. DH376 was dissolved in ethanol-PEG-saline 0.9% (1:1:18, v/v/v). Sumatriptan succinate (0.6 mg/kg, dissolved in saline) was dosed subcutaneously 30 min before the injection of DAGL inhibitor. Olcegepant 0.8 mg/kg, dissolved in DMSO-Tween80-saline 0.9% (1:1:8, v/v/v) was injected intraperitoneally 30 min before the administration of DAGL inhibitor. AEA-d4 and 2-AG-d5 were purchased from Cayman Chemicals (Ann Arbor, MI).

### Animals

Female and male Sprague Dawley rats (7–8 weeks old) were purchased from Envigo (Indianapolis, IN) and housed in a climate-controlled room on a regular 12/12 h light/dark cycle with lights on at 7:00 am with food and water available *ad libitum*. Animals were housed three per cage. All procedures were performed during the 12-h light cycle and according to the policies and recommendations of the International Association for the Study of Pain and the NIH guidelines for laboratory animals, and with IACUC approval from the University of Arizona. Justification for animal numbers was consistent with NIH policy (NOT-OD-15-102), and experiments were randomized to blinded treatment groups, giving 80% power to detect a treatment effect size of 20% compared to a baseline response of 5% at a significance level of 0.05 (Andrews et al., 2016). Numbers required to achieve statistical power were determined by G.Power3.1.

### Medication Overuse Headache (MOH) Induced by Sustained Sumatriptan or Morphine Infusion

Alzet osmotic minipumps (Alzet, Cupertino, CA, United States; model 2001) with a flow rate of 1 μL/h for 7 days were used in female rats for drug infusion, as described in previous reports (De Felice et al., 2010; Bonnet et al., 2019). The minipumps were implanted subcutaneously under anesthesia with isoflurane (flow rate = 2 L/min in O_2_). A 4–5 mm incision was made between the shoulder blades. The osmotic minipump was inserted under the skin. The incision site was closed with wound clips. The day of the pump implant was considered as day 0. Drugs administered by infusion were either sumatriptan (0.6 mg/kg/day) or morphine (5 mg/kg/day) or vehicle (NaCl, 0.9%). On day 7, spatially discrete brain regions (Ct, occipital cortex; PAG, periaqueductal gray; Vc, trigeminal nucleus caudalis; and TG, trigeminal ganglia) were harvested, as described below (“*Harvest of Tissue Samples*” Section).

### Assessment of Periorbital and Hind-Paw Mechanical Allodynia

Mechanical allodynia was evaluated before and at 30, 60, 90, 120, 180, 360 min, and 24 h after drug treatment by an observer blinded to drug administration. Any rats exhibiting excessive allodynia at baseline (<75% of cut-off value; threshold <6 g for periorbital or <11.25 g for hind-paw) were removed from the study. Rats were acclimated to testing box 1 hour prior to evaluation of mechanical allodynia with calibrated von Frey filaments as previously described by Edelmayer and coworkers (Edelmayer et al., 2012). Behavioral responses of periorbital sensitivity were determined by applying calibrated von Frey filaments perpendicularly to the midline of the forehead at the level of the eyes with enough force to cause the filament to slightly bend while held for 5 s. A response was indicated by a sharp withdrawal of the head, vocalization, or severe batting at the filament with attempts to eat it. Hind-paw withdrawal thresholds were tested by perpendicular application of the filaments to the plantar surface of the left hind-paw. The withdrawal threshold was calculated using a modified version of the Dixon up-down method (Dixon, 1980).

### Open Field Test

Open field test was performed 2 h post-injection of LEI106 to assess anxiety-associated behaviors. The open-field arena (90 cm × 90 cm × 40 cm) is a white box with an open top and a black floor. The test started by placing the animal in the center of the arena and allowing free movement for a 5 min duration. A consistent white noise (∼55 dB) and a dim lighting (∼24 lux) were applied during the test. The behavior of each rat was analyzed in real-time using a digital web camera mounted 1.5 m above the floor in conjunction with Any-Maze software (version 4.75, Stoelting, Wood Dale, IL), a video tracking system designed to automate testing in behavioral experiments. Using the software, the arena was divided into a scaled grid of equally sized squares spaced at 10 cm, resulting in a total of 28 squares. Eight of the inner squares were assigned as the center zone, while the remaining twenty were assigned as the perimeter zone. The main parameters analyzed were total distance traveled and the amount of time spent in each of the zones.

### Light-Dark Box Test

Light-dark box test was conducted following the open-field test. For the light-dark box test (LDBT), a 3-compartment place preference box (San Diego Instruments, San Diego, CA) was utilized. This system consists of three adjoined chambers; one of the end chambers (27 cm × 21 cm × 34 cm) was covered with opaque black vinyl so that no light would pass through, while the opposing end chamber (27 cm × 21 cm × 34 cm) was open and illuminated (40 W); the center chamber (14 cm × 21 cm × 34) contained a 7 cm opening on either end to allow the animals to pass from one end to the other, it was also unlit and served as a transition zone to further remove the dark chamber from the light source. A 4 × 16 photobeam array runs along the bottom of each box; the beams are broken as the animal travels from one compartment to the other. The software (PAS Data Reporter, version 1.0.2.7, San Diego Instruments, San Diego, CA) which is run with these boxes records the amount of time the animal spends in each compartment by tracking the path and number of beam breaks. Each animal was placed into the center compartment and allowed to move freely for 15 min. The main parameters were duration of time spent in the light box; the latency to enter the dark box; and the number of transitions between the light and dark box.

### Elevated Plus Maze (EPM) Anxiety Test

Immediately following the LDBT, elevated plus maze test was conducted, which can measure many relevant anxiety related behaviors such as, number of pokes and full entries into and duration spent in closed vs. open arm. The EPM consists of four elevated arms (50 cm long and 10 cm wide) with two opposing arms containing 30 cm high opaque walls. The arms and central platform of the apparatus are elevated to a height of 62 cm. Each rat was allowed 5 min to explore the EPM and then returned to its home cage. Between animals the EPM was cleaned thoroughly with Versa-Clean (Fisher Scientific). EPM performance was video recorded for later analyses using ANYmaze software (version 4.75, Stoelting, Wood Dale, IL). The main parameters were duration of time spent in the open and closed arm and number of entries into the arms.

### Harvest of Tissue Samples

Spatially discrete brain regions (occipital cortex-Ct, periaqueductal gray-PAG, trigeminal nucleus caudalis-Vc, and trigeminal ganglia-TG), involved in pain signaling were harvested 2 h after the injection of LEI106 (20 mg/kg, ip.) or KT109 (20 mg/kg, ip.), along with vehicle-treated controls. The animals were anesthetized with ketamine:xylazine mix (80:10 mg/kg, ip.), then transcardially perfused with ice cold 0.1 M phosphate buffer at rates to not burst brain microvasculature (i.e., 3.1 ml/min). After decapitation, tissue samples were harvested, flash frozen in liquid nitrogen and stored at −80°C until further application.

### Quantification of 2-AG and AEA by LC-MS

The brain samples for LC-MS were purified by organic solvent extraction, as described by Wilkerson et al. (Wilkerson et al., 2016). Briefly, tissues were harvested, snap-frozen in pre-weighted tubes, and stored at −80°C. On the day of processing, tissues were weighed and homogenized in 1 ml of chloroform/methanol (2:1 v/v) supplemented with phenylmethylsulfonyl fluoride (PMSF) at 1 mM final concentration to inhibit the degradation by endogenous enzymes. Dounce homogenizer was used for homogenization. Homogenates were then mixed with 0.3 ml of 0.7% w/v NaCl, vortexed, and then centrifuged for 10 min at 3,200 × g at 4°C. The aqueous phase plus debris were collected and extracted two more times with 0.8 ml of chloroform. The organic phases from the three extractions were pooled and internal standard was added to each sample. Mixed internal standard solutions were prepared by serial dilution of AEA-d4 and 2-AG-d5 in 80% acetonitrile. The organic solvents were evaporated under nitrogen gas. 6 uL of 30% glycerol in methanol per sample was added before evaporation. Dried samples were reconstituted with 0.2 ml of chloroform and mixed with 1 ml of ice-cold acetone to precipitate proteins. The mixtures were then centrifuged for 5 min at 1,800 × g at 4°C. The organic layer of each sample was collected and evaporated under nitrogen.

Analysis of 2-AG and AEA was performed on an Ultivo triple quadrupole mass spectrometer combined with a 1290 Infinity II UPLC system (Agilent, Palo Alto, CA). The instrument was operated in electrospray positive mode with a gas temperature of 150°C at a flow of 5 L/min, nebulizer at 15 psi, capillary voltage of 4,500 V, sheath gas at 400°C with a flow of 12 L/min and nozzle voltage of 300 V. Transitions monitored were 348.3 → 287.3 and 62, 352.3 → 287.4 and 65.9, 379.3 → 287.2 and 269.2, and 384.3 → 287.2 and 296.1 for AEA, AEA-d4, 2-AG, and 2-AG-d5. The first fragment listed was used for quantification and the second fragment was used for confirmation. The first 3 min of analysis time was diverted to waste. Chromatographic separation was achieved using an isocratic system of 21% 1 mM ammonium fluoride and 79% methanol on an Acquity UPLC BEH C-18 1.7u 2.1 × 100 mm column (Waters, Milford, MA) maintained at 60°C. After each injection the column was washed with 90% methanol for 1 min then re-equilibrated for 5 min prior to the next injection. Samples were maintained at 4°C. Mixed calibration solutions were prepared by serial dilution of AEA and 2-AG stock solutions in 80% acetonitrile. Calibration curves were prepared for each analysis by adding 10 µL internal standard solution to 20 µL standard solution. Prior to sample analysis, 200 µL of 80:20 acetonitrile:water was added to dried samples which were then vortexed and sonicated. The samples were centrifuged at 15,800 × g at 4°C for 5 min. Supernatant was transferred to autosampler vials and 5 µL was injected for analysis.

### Quantification of Diacylglycerol (DAG) by ELISA

Quantification of diacylglycerol (DAG) ELISA kit (Aviva System Biology, OKEH02607) was used according to manufacturer’s instruction. Briefly, tissue samples were weighed, homogenized in 1× PBS buffer and stored overnight at ≤−20°C. Two freeze-thaw cycles were performed to break the cell membranes then homogenates were centrifuged at 5,000 × g at 4°C for 10 min. 5 µL of the supernatant was applied in the immunoassay.

### Data Analysis and Statistics

GraphPad Prism 7.0 software (GraphPad Software) was used for statistical analysis. Unless otherwise stated, the data were expressed as mean ± SEM. Mechanical allodynia measurements were assessed using a repeated measure two-way ANOVA to analyze differences between treatment groups over time with either a Bonferroni or Tukey test applied post hoc. Molecular studies were compared by Student’s *t*-test or one-way ANOVA, as indicated. Differences were considered significant if *p* ≤ 0.05 to give 80% power to detect at 20% difference and prevent a type II error (GPower3.1).

## Results

### 2-AG, AEA Levels in Medication Overuse Headache Models

**FIGURE 1:**
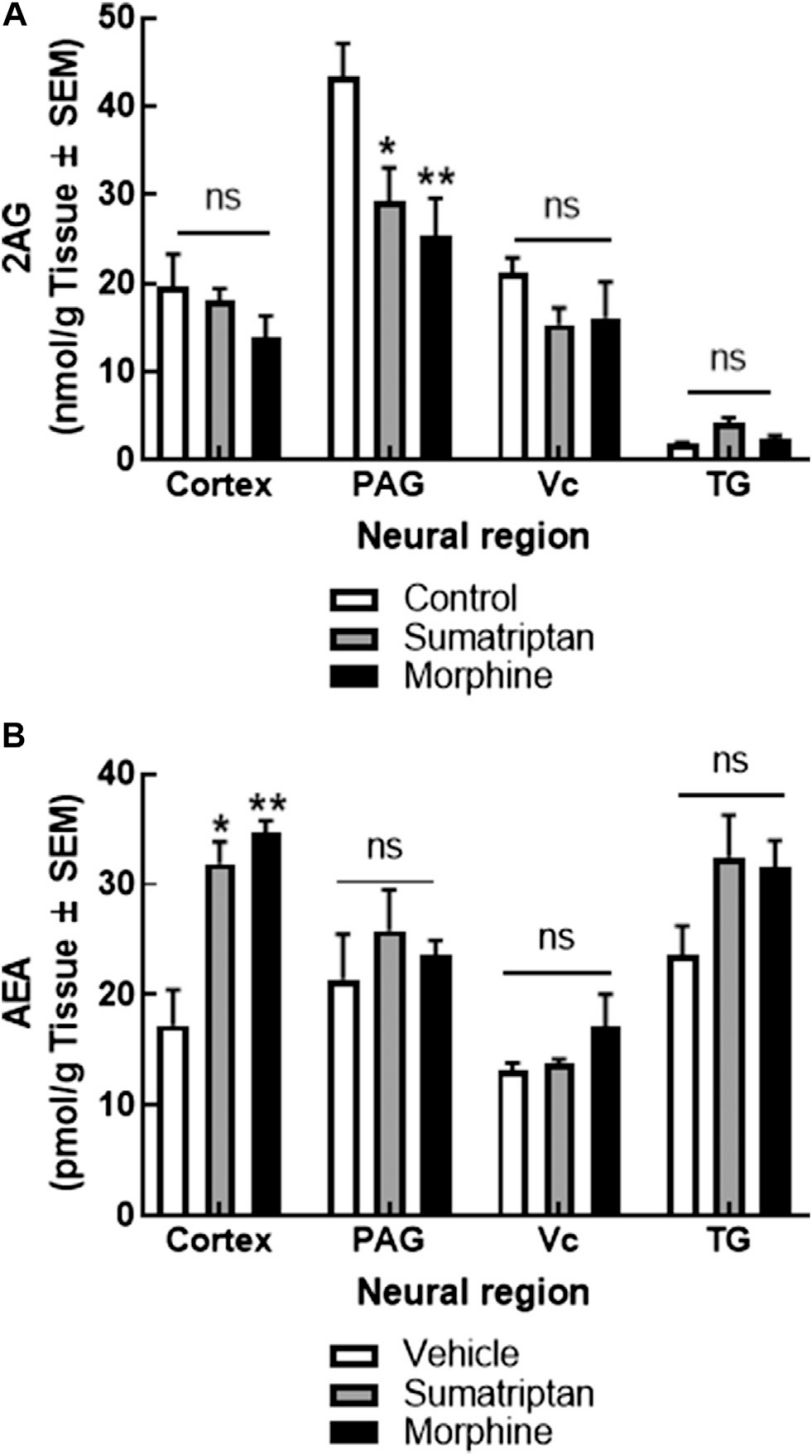
Sustained administration of sumatriptan or morphine, as models of medication overuse headache, changed eCB levels in the cortex and PAG. 2-AG levels in the PAG, but not cortex, Vc, or TG were reduced after 7 days of sumatriptan (0.6 mg/kg/day, SC) or morphine (5 mg/kg/day, SC) infusion by osmotic minipump in female rats. **(A)** AEA levels were increased in the cortex, but not PAG, Vc, or the TG, in the same animals. **(B)** Data are expressed mean ± SEM (n = 3–9/group), analyzed by two-way ANOVA, *p < 0.05, **p < 0.01, ns, non-significant.

Endocannabinoid deficiency is primarily reported in chronic migraine patients (Burston and Woodhams 2014; McPartland et al., 2014; Gouveia-Figueira et al., 2017). To determine if this observation was recapitulated in preclinical medication overuse headache with regional selectivity in the CNS, female rats were continuously administered with either sumatriptan (0.6 mg/kg/day) or morphine sulfate (5 mg/kg/day) via osmotic minipump for 7 days. Following tissue harvest on D7, levels of 2-AG and AEA were determined in the V1M cortex (cortex), PAG, Vc, and TG using LC-MS (Figure 1). Mean levels of AEA in tissues for control (vehicle-treated) animals were as follows in pmol/g tissue: cortex: 17.24 ± 3.20, PAG: 21.43 ± 4.06, Vc: 13.12 ± 0.74, and TG: 23.64 ± 2.61. Chronic infusion of both sumatriptan and morphine for 7 days significantly increased levels of AEA in the cortex without changing levels in the PAG, Vc, or TG (two-way ANOVA F(2,51) = 4.37; *p* = 0.017; Tukey post-hoc *p* = 0.031 and *p* = 0.004, respectively) when compared to chronic vehicle treated animals.

Quantified mean control levels of 2-AG in nmol/g tissue were: cortex: 19.16 ± 3.64, PAG: 43.31 ± 3.85, Vc: 21.17 ± 1.73, and TG: 1.80 ± 0.17. Chronic infusion of both sumatriptan and morphine significantly reduced 2-AG levels in the PAG without changing levels in the cortex, Vc, or TG (Two-way ANOVA F(2,48) = 6.80; *p* < 0.003; Tukey post-hoc *p* = 0.018 and *p* = 0.004, respectively) when compared to chronic vehicle infusion. Thus, sustained administration of two analgesics associated with medication overuse headache altered eCB levels. Since clinically, endocannabinoid deficiency is associated with headache, the next experiments focused on recapitulating 2-AG depletion.

### Selective Blockade of DAGLα, but Not DAGLβ, Induced Facial Allodynia Without Causing Peripheral Sensitivity

**FIGURE 2:**
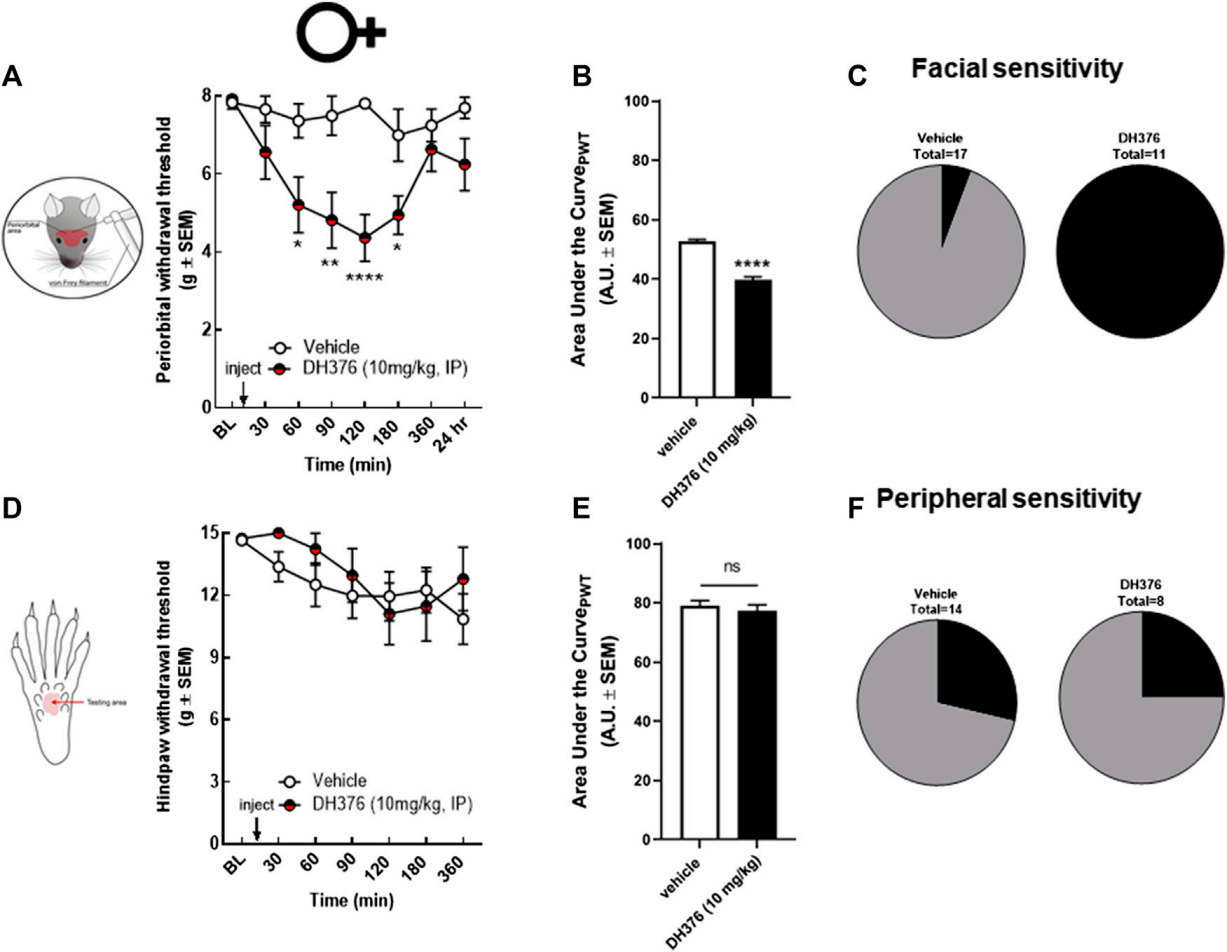
Non-selective inhibition of DAGL induced periorbital but not peripheral mechanical allodynia in female rats. Intraperitoneal injection of DH376 (10 mg/kg, IP) significantly decreased periorbital withdrawal threshold **(A)** at 60, 90, 120, and 180 min post-injection compared to vehicle-treated controls. Data are expressed mean ± SEM (n = 11–17/group), analyzed by two-way ANOVA, *p < 0.05, **p < 0.01, ****p < 0.0001. Hind-paw withdrawal thresholds after injection of DH376 were not significantly different compared to vehicle-treated controls **(D)**. Data are expressed mean ± SEM (n = 8–14), analyzed by two-way ANOVA, ns, non-significant. The area under the curve for the corresponding time-course experiments **(B,E)**, assessed by Student’s *t*-test, ****p < 0.0001, ns, non-significant. The percentage of animals in vehicle-, and in DAGL-inhibitor treated groups, showing facial **(C)** and peripheral **(F)** sensitivity, as defined FWT < 6 g at two consecutive time-points in facial area.

To investigate the possible connection between endocannabinoid depletion and headache-like pain, we tested the effect of a non-selective DAGL inhibitor, DH376, in female rats. DH376 was intraperitoneally injected at 10 mg/kg dose. The ED50 value of DH376 after intraperitoneal injection was determined between 5–10 mg/kg, showing blockade of DAGLα and DAGLβ in mice (Ogasawara et al., 2016). The periorbital and peripheral mechanical allodynia was assessed before and at 30, 60, 90, 120, 180, 360 min, and 24 h after drug treatment by calibrated von Frey filaments. DH376 (10 mg/kg, ip) significantly decreased periorbital withdrawal threshold at 60, 90, 120, and 180 min post-injection compared to vehicle-treated controls (Figures 2A,B; DH376 vs. vehicle, *p* = 0.01 at 60 min, *p* = 0.013 at 90 min, *p* < 0.0001 at 120 min, and *p* = 0.02 at 180, two-way ANOVA with Tukey’s post-test, n = 11–17/group, F(7,160) = 2.648); (AUC- DH376: 39.61 ± 3.82 vs. vehicle: 52.67 ± 2.39, *p* < 0.0001, Student’s *t*-test). All DH376-treated animals showed facial sensitivity, which was defined as FWT < 6 g at two consecutive time-points (Figure 2C). The treatment of non-selective DAGL inhibitor did not cause significant changes in hind-paw withdrawal threshold (Figures 2D–F; AUC- DH376: 78.93 ± 5.51 vs. vehicle: 77.16 ± 6.33, *p* = 0.53, Student’s *t*-test), suggesting that loss of DAGL activity plays a role in facial, but not hind-paw, mechanical allodynia.

**FIGURE 3:**
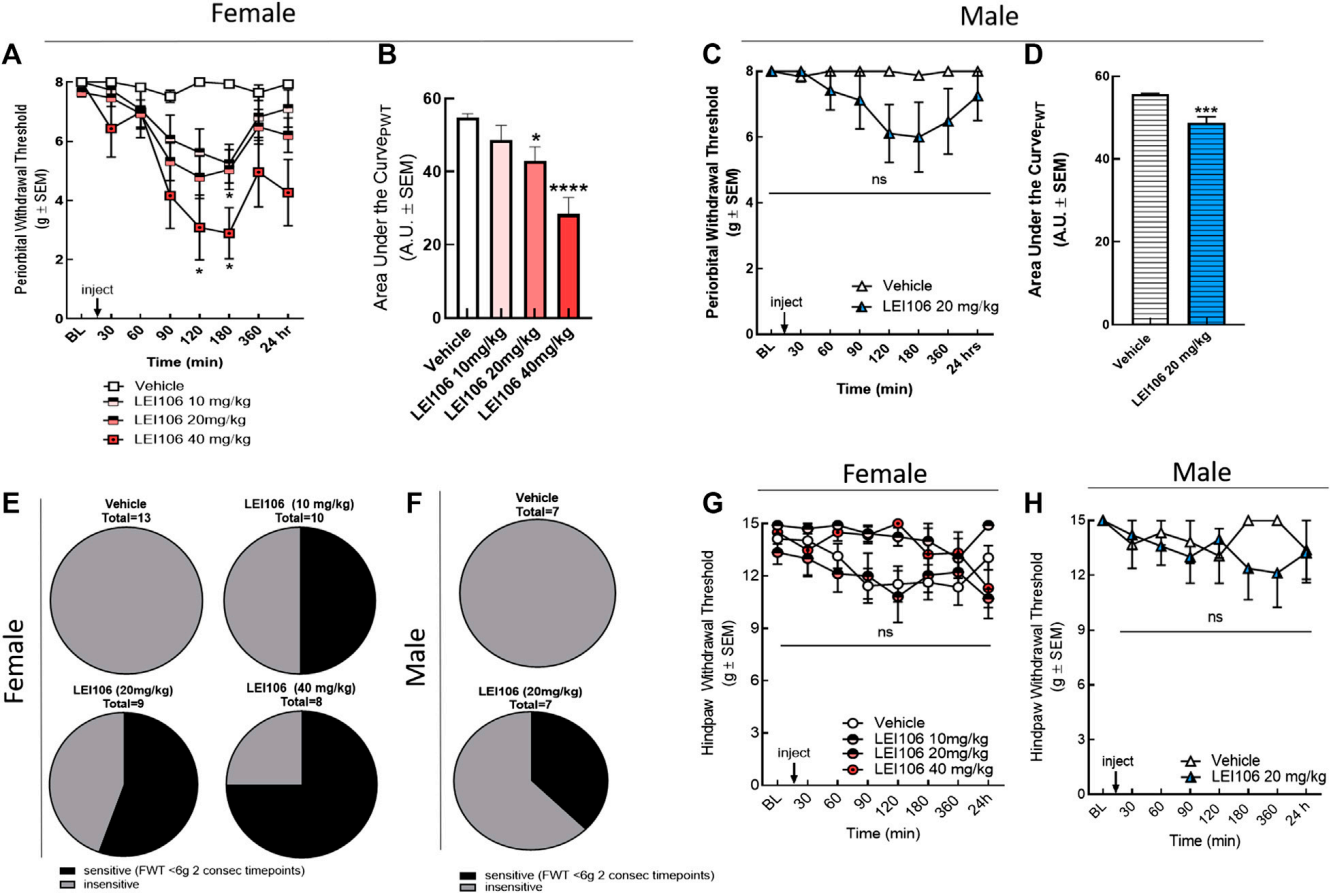
DAGLα inhibition with LEI106 induced significant periorbital but not hind-paw allodynia. Intraperitoneal injection of LEI106 at 10, 20, and 40 mg/kg induced facial allodynia in female animals **(A)**. LEI106 at 20 and 40 mg/kg doses caused significant reduction in facial withdrawal thresholds at 120 and 180 min post-injection compared to vehicle control. Data are expressed mean ± SEM (n = 8–13), two-way ANOVA, *p < 0.05, ***p < 0.001, ****p < 0.0001. In male animals, intraperitoneal injection of LEI106 at 10 mg/kg did not cause significant changes in facial withdrawal thresholds in any time-point **(C)**. Data are expressed mean ± SEM (n = 7), two-way ANOVA, ns, non-significant. The area under the curve for the corresponding time-course experiments **(B,D)**, assessed by one-way ANOVA (female) or Student’s *t*-test (male), **p < 0.01, ***p < 0.001, ****p < 0.0001. The percentage of animals in vehicle-, and in LEI106-treated groups, showing facial sensitivity **(E,F)**, as defined FWT < 6 g at two consecutive time-points in facial area. LEI106 did not significantly influence the hind-paw withdrawal thresholds in either doses in either sexes **(G,H)**, suggesting the absence of peripheral action.

Next, the contributions of the individual DAGL isoforms to cephalic vs. hind-paw allodynia was tested. Female rats were treated with selective DAGL inhibitors, and the mechanical allodynia was evaluated before and at 30, 60, 90, 120, 180, 360 min, and 24 h after drug treatment in facial and left hind-paw, as described above. Selective inhibition of DAGLα, by LEI106 induced facial allodynia (Figure 3). LEI106 at 20 and 40 mg/kg significantly reduced periorbital withdrawal thresholds 120 and 180 min post-injection as compared to vehicle control (Figure 3A) (two-way ANOVA with Bonferroni post-test, n = 9–13/group, Ftime x treatment(21, 284) = 3.472; *p* = 0.017–0.039). The 10 mg/kg LEI dose reduced periorbital withdrawal thresholds by ∼35% 120- and 180-min post administration; this did not reach statistical significance (*p* = 0.07 and *p* = 0.06, respectively; Figure 3A). Moreover, the highest dose of LEI106 showed the longest duration of effect, the facial threshold was significantly reduced at 24 h time-point after the injection of LEI106 (Figure 3A). While magnitude was similar between 10 and 20 mg/kg, escalating doses of LEI106 increased the percentage of animals exhibiting facial sensitivity, defined as FWT < 6 g at two consecutive time-points (Figure 3E). Calculation of the area under the curves revealed that LEI106 induced significant dose-dependent periorbital allodynia (Figure 3B, one-way ANOVA F = 10.85, *p* < 0.001; Tukey post-hoc *p* = 0.036 and *p* < 0.0001).

To assess sex as a behavioral variable in this model, male rats were injected with calculated A50 dose from females of LEI106 (20 mg/kg, IP) and evaluated for periorbital and hind-paw allodynia over time (Figures 3C,D,F,H). LEI106 (20 mg/kg) induced significant periorbital allodynia in 3/8 rats (FWT < 6 g at two consecutive timepoints (Figure 3F). Collectively, facial withdrawal thresholds were reduced by 24 and 25% of baseline at 120 and 180 min, respectively; this was not statistically significant (n = 8, two-way ANOVA, Ftime x treatment(7,89) = 1.913, *p* = 0.077, Bonferroni post-hoc). Area under the curve analysis showed a significant reduction in facial threshold over time (Figure 3D, Student’s *t*-test *p* = 0.0006).

To determine if DAGLα inhibition induced peripheral sensitivity, hind paw allodynia was assessed. Similar to the non-selective blockade of DAGL, selective inhibition of DAGLα did not induce hind-paw sensitivity in female rats at any dose tested as compared to vehicle (Figure 3G) (two-way ANOVA Ftime x treatment(18,209) = 0.845, *p* = 0.65; Tukey post-hoc; n = 8–13). Hind-paw allodynia was observed in 2/8 male rats (two consecutive timepoints <12 g), but overall, no significant hind-paw allodynia was observed in the cohort (n = 7, two-way ANOVA, Ftime x treatment(7,91) = 1.427, *p* = 0.20; Figure 3H). Periorbital allodynia was not observed in vehicle control animals of either sex (Figures 3E–H).

**FIGURE 4:**
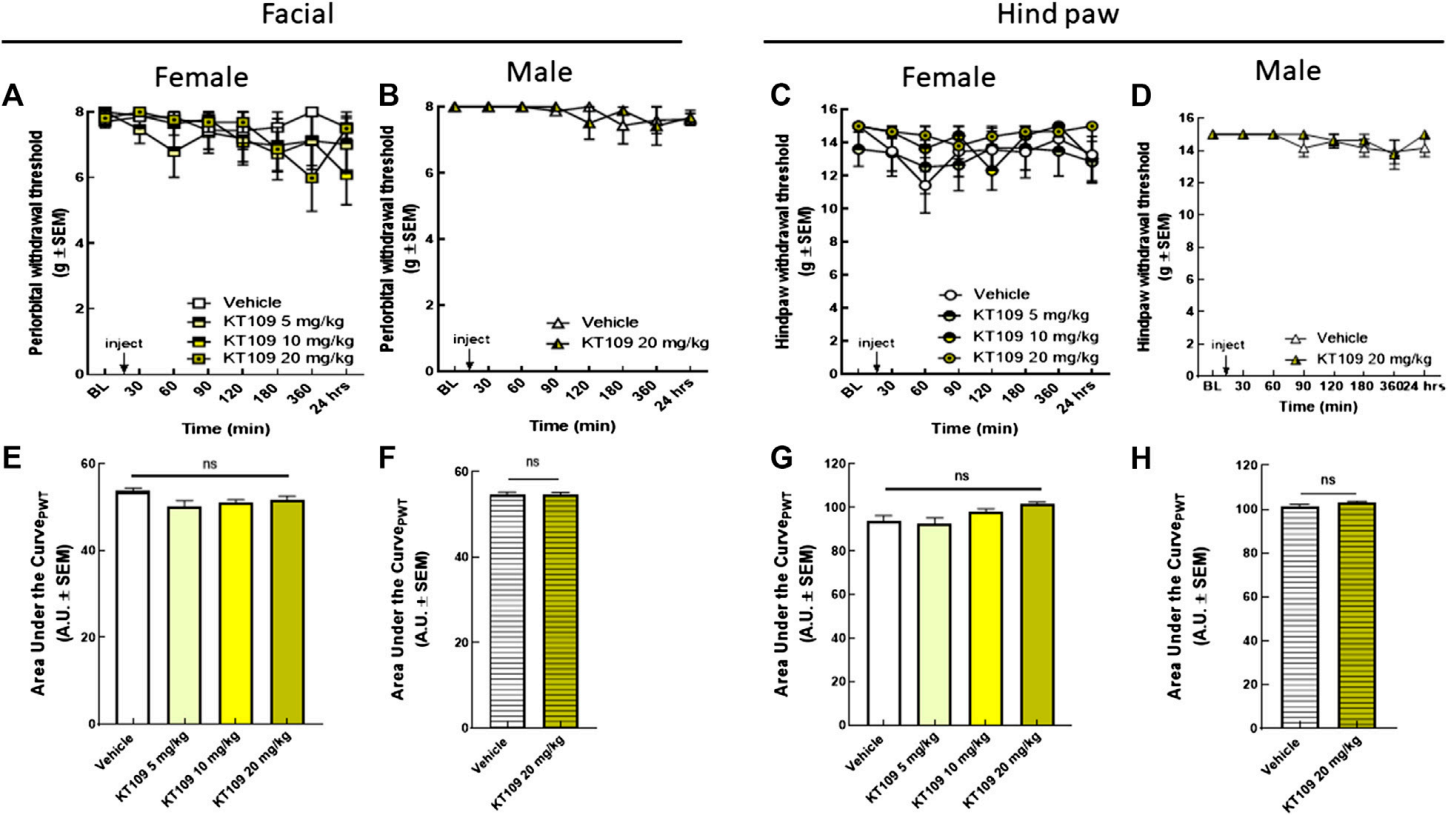
Inhibition of DAGLβ did not induce periorbital or hind-paw allodynia in either sex. Injection of KT109 (5, 10, 20 mg/kg, IP) did not significantly influence periorbital **(A)** or hind-paw withdrawal thresholds **(C)** in female rats. Data are expressed mean ± SEM (n = 6–9), two-way ANOVA, ns, non-significant. Intraperitoneal injection of KT109 (20 mg/kg) did not cause significant changes of facial **(B)** or hind-paw **(D)** thresholds in male animals. Data are expressed mean ± SEM (n = 8), two-way ANOVA, ns, non-significant. The area under the curve for the corresponding time-course experiments **(E–H)**, assessed by one-way ANOVA or Student’s *t*-test, ns, non-significant.

KT109, a selective inhibitor, was used to assess DAGLβ contributions to the development of periorbital allodynia (Figure 4). Increasing doses of KT109 (5, 10, 20 mg/kg, IP) did not induce significant periorbital (Figure 4A) or hind paw allodynia (Figure 4C) across the experimental duration in female rats. Dosing of male rats with KT109 (20 mg/kg) showed similar results (Figures 4B,D). Together, these data indicate that inhibition of DAGLα, but not DAGLβ, is enough to induce cephalic, but not extracephalic, mechanical sensitivity with a pronounced effect in females.

### Periorbital Allodynia Induced by DAGLα Inhibition Is Sensitive to Anti-Migraine Agents

**FIGURE 5:**
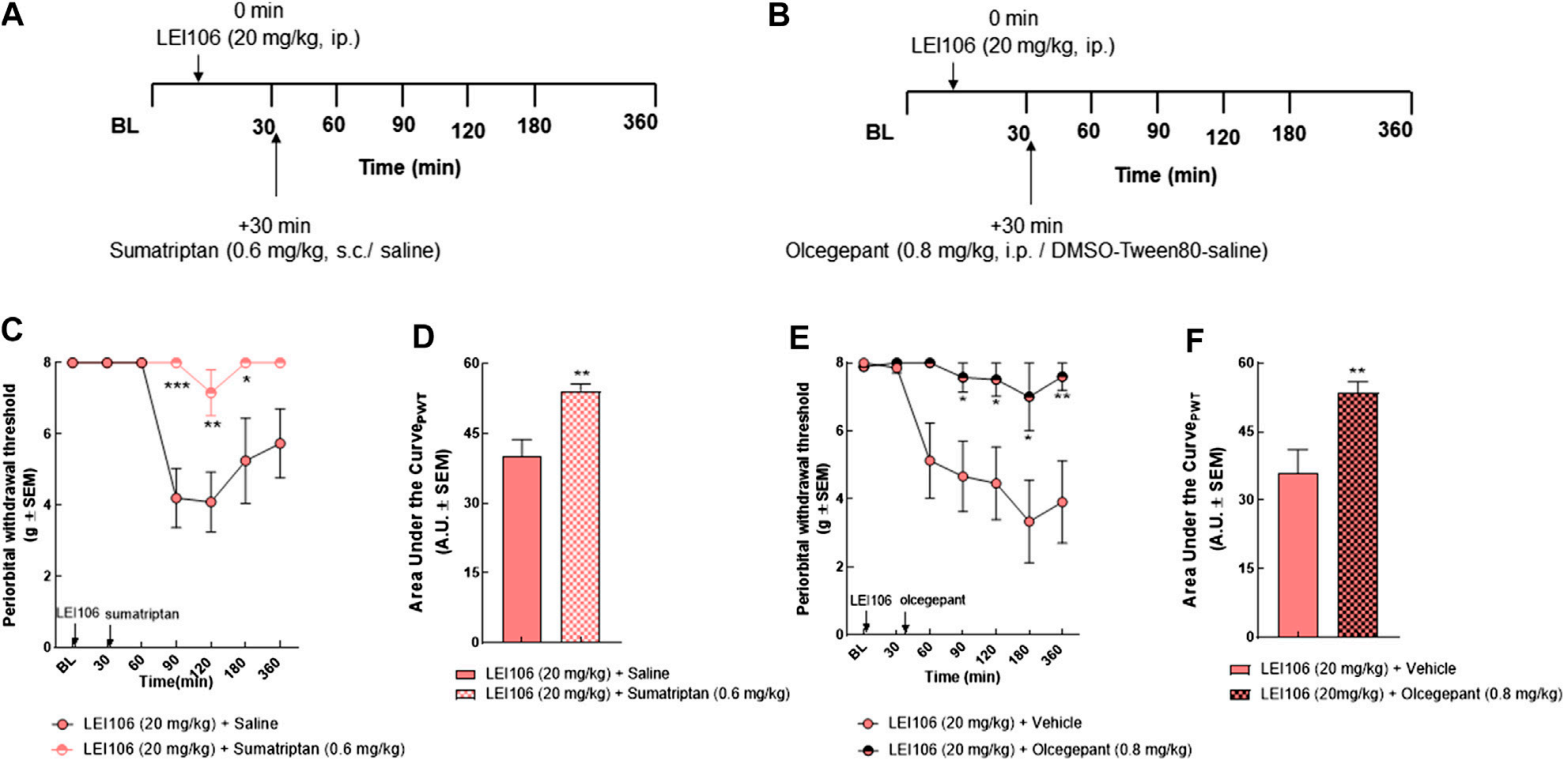
Periorbital allodynia induced by DAGLα inhibition is sensitive to anti-migraine agents, sumatriptan and olcegepant. Timeline of experiments **(A,B)**. Injection of sumatriptan (0.6 mg/kg, SC) significantly mitigated the periorbital allodynia induced by LEI106 (20 mg/kg, IP) at 90, 120, and 180 min after dosing **(C)**. Data are expressed mean ± SEM (n = 8), two-way ANOVA, *p < 0.05, **p < 0.01, ***p < 0.001. Administration of olcegepant (0.8 mg/kg, IP) significantly attenuated the periorbital allodynia induced by LEI106 (20 mg/kg, IP) at 90, 120, 180, and 360 min after dosing **(E)**. Data are expressed mean ± SEM (n = 8), two-way ANOVA, *p < 0.05, **p < 0.01. The area under the curve for the corresponding time-course experiments **(D,F)**, assessed by Student’s *t*-test, **p < 0.01.

Responsivity to the acute dosing of abortive antimigraine agents, sumatriptan and olcegepant were used to validate DAGLα inhibition as a non-invasive, preclinical model of headache. Female rats were treated with LEI (20 mg/kg, IP) and the mechanical allodynia allowed to develop. Thirty minutes after LEI106 dosing, female rats were injected with either sumatriptan (0.6 mg/kg, SC) or olcegepant (0.8 mg/kg, IP) and facial mechanical sensitivity reassessed (Figure 5). As in Figure 3, LEI + saline induced significant periorbital allodynia in 5/7 rats. Sumatriptan significantly mitigated this allodynia 90, 120, and 180 min after dosing in 6/7 rats (Figures 5C,D) (LEI106 + sumatriptan vs. LEI106 + saline, two-way ANOVA with Tukey’s post-test, n = 8/group, F(6,61) = 4.439, *p* = 0.0009). Responsivity to the CGRP receptor antagonist was assessed in a separate set of rats (Figures 5E,F). LEI106 + vehicle (10%DMSO: 10% Tween80: 80% saline) induced significant periorbital allodynia in 5/8 rats. Olcegepant dosed after DAGLα significantly attenuated the periorbital allodynia associated with LEI106 at 90, 120, 180 and 360 min after LEI dosing; allodynia was observed in 1/8 rats (LEI106 + olcegepant vs. LEI106 + saline, two-way ANOVA with Tukey’s post-test, n = 8/group, F(6,74) = 3.021, *p* = 0.011). These results suggest that periorbital allodynia induced by DAGLα inhibition is reversed by two clinically relevant, antimigraine agents.

### DAGLα Inhibition Induces Non-Allodynic Measures of Pain Linked to Headache but Not Anxiety

**FIGURE 6:**
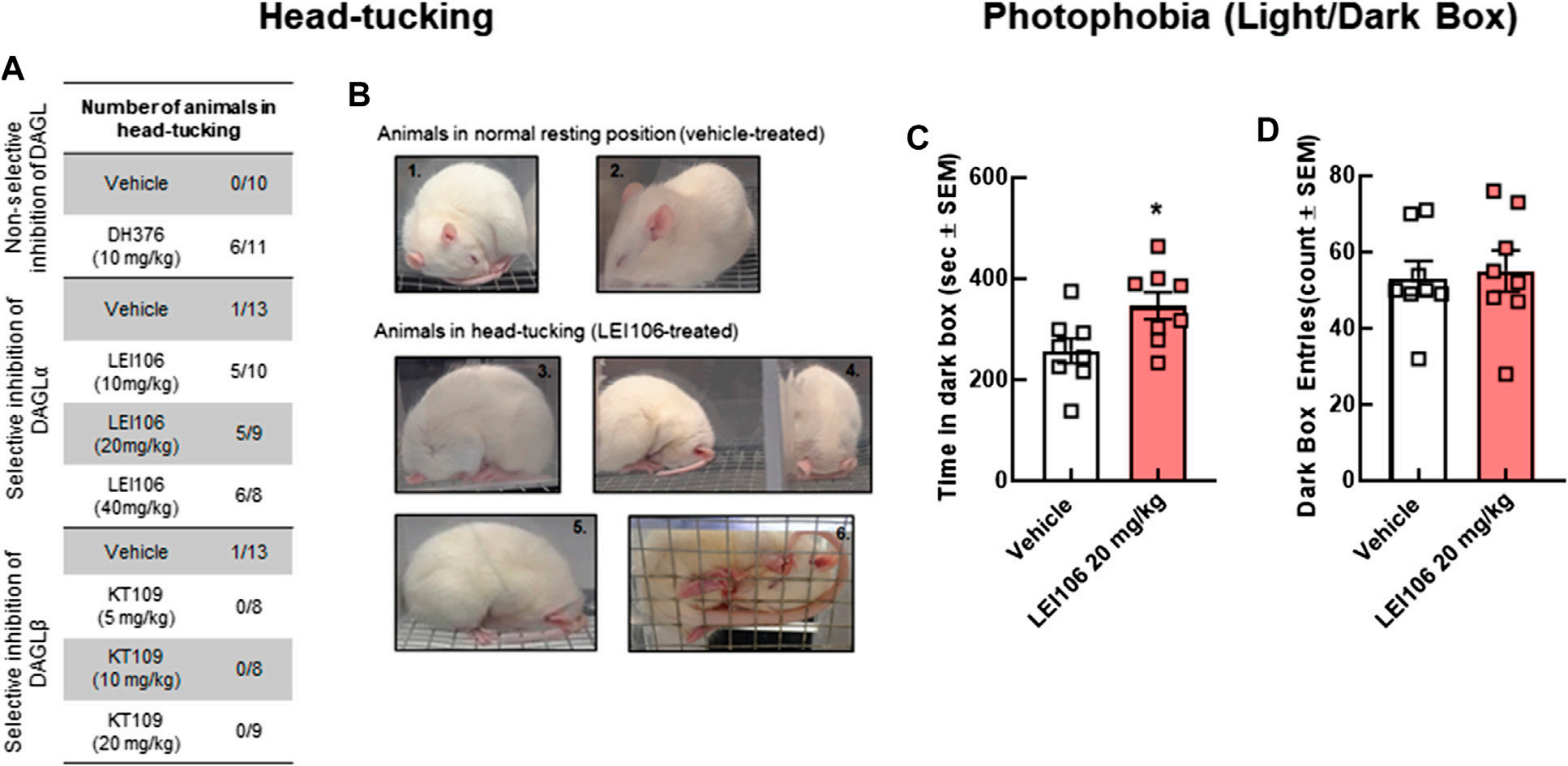
DAGLα inhibition induces non-allodynic measures of pain linked to headache in female rats. The animals treated with non-selective inhibitor, DH376 (10 mg/kg, IP) or DAGLα-selective, LEI106 (10, 20, and 40 mg/kg, IP) showed spontaneous pain-related behavior, known as head-tucking. After injection of DAGLβ inhibitor, KT109, head-tucking behavior was not observed. The number of animals, showing head-tucking/total number of animals in each treatment group **(A)**, representative photographs about animals in normal resting position **(B1,B2)** after vehicle-injection, and in head-tucking-position **(B3–B6)** after treatment with LEI106. In light-dark box experiment, female rats administered LEI106 (20 mg/kg, IP) spent significantly more time in the dark chamber as compared to the light side **(C)**. However, there was no significant differences in the number of entries **(D)**. Data are expressed mean ± SEM (n = 8), Student’s *t*-test, *p < 0.05, ns, non-significant.

Head pressing against a hard surface is a sign of neurological dysfunction in animals and is linked to severe pain in rodents (Kohn et al., 2007). Administration of LEI106 dose-dependently increased the number of animals exhibiting head-pressing behaviors (Figures 6A,B). Neither vehicle, nor the DAGLβ induced head pressing behaviors after systemic dosing.

In addition to facial sensitivity, migraine headache is associated with photophobia. To determine if DAGLα inhibition induced sensitivity to light, rats could choose between light and dark environments in the light/dark preference assay at the time of peak periorbital allodynia, 120 min post-injection. Female rats administered LEI106 (20 mg/kg, IP) spent significantly more time in the dark chamber as compared to the light despite the number of entries being the same (Figure 6C; LEI106 vs. vehicle, Student’s *t*-test *p* = 0.03, n = 8/group).

**FIGURE 7:**
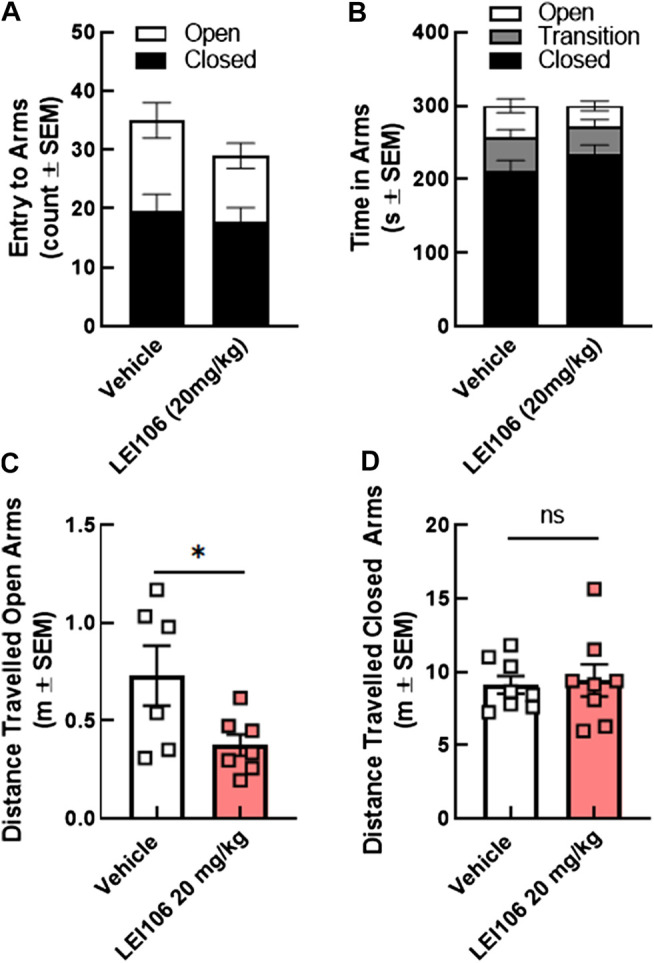
DAGLα inhibition did not induce anxiety in the elevated plus maze tests in female rats. LEI106 (20 mg/kg, IP) did not statistically influence the number of entries between the open, closed and transition square when compared to vehicle treated animals **(A)**. No statistically significant differences between vehicle and LEI106 treatments were observed in the total time in each arm **(B)**. However, administration of LEI106 (20 mg/kg, IP) significantly reduced the total distance traveled in the open arms, but not closed **(C,D)**. Data are expressed mean ± SEM (n = 8), Student’s *t*-test, *p < 0.05, ns, non-significant.

Given that the light-dark box is used for both photophobia and anxiety measures (Albani et al., 2015), the same rats were assessed for anxiety like behaviors using the elevated plus maze. LEI106 (20 mg/kg, IP) did not statistically change the number of entries between the open, closed and transition square when compared to vehicle treated animals (Figure 7A); the total time in each arm was also similar between vehicle and LEI106 treatments (Figure 7B). LEI106 administration (20 mg/kg, IP) significantly reduced the total distance traveled in the open arms, but not closed (Figures 7C,D; Student’s *t*-test, n = 8/group, *p* = 0.04).

**FIGURE 8:**
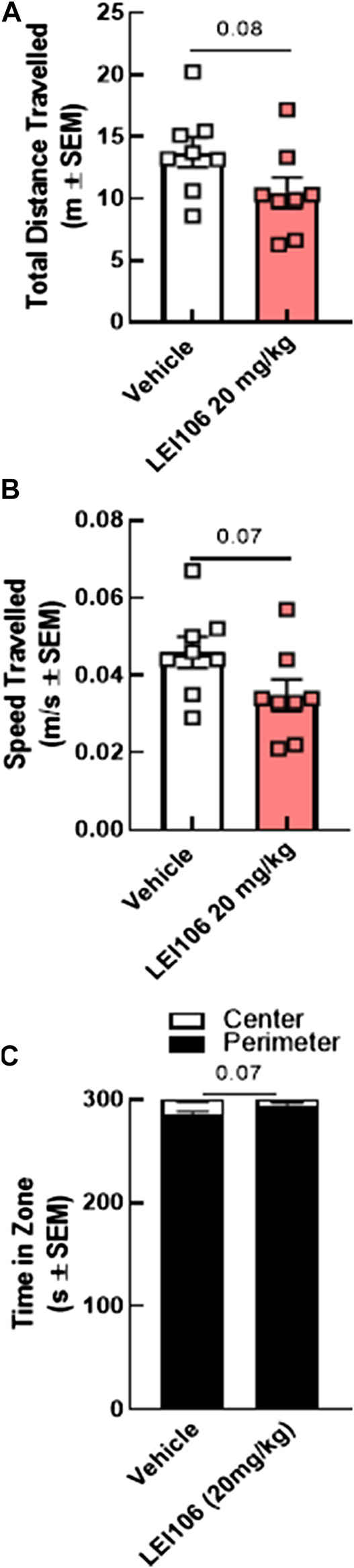
DAGLα inhibition did not influence voluntarily movements and motor skills in the open field tests in female rats. The injection of LEI106 (20 mg/kg IP) did not statistically change the total distance traveled **(A)**, the sped of travel **(B)**, or the time spent in the center vs. the perimeter zone **(C)**. Data are expressed mean ± SEM (n = 8), Student’s *t*-test, ns, non-significant.

Several headache models have reported changes in open field parameters as a measure of motor skill, moreover, the ability to choose a preferred environment relies on the ability to move voluntarily (Vuralli et al., 2019). To assess whether reduced movements in the light-dark assay and elevated plus maze were confounds by reduced movement, rats were evaluated using the open field test. LEI106 dosing did not statistically change the total distance traveled, sped of travel, or time spent in the center vs. the perimeter zone (Figure 8; LEI106 vs. vehicle, total distance traveled: *p* = 0.08, speed of travel: *p* = 0.07; time spent in the center vs. the perimeter zone: *p* = 0.07, Student’s *t*-test, n = 8/group). Together, these data suggest DAGLα inhibition produces migraine-like behaviors of allodynia and photophobia without reducing voluntary movement or acting as an anxiogenic.

### Regional Differences in 2-AG, AEA, but Not DAG Levels After DAGL Inhibition

**FIGURE 9:**
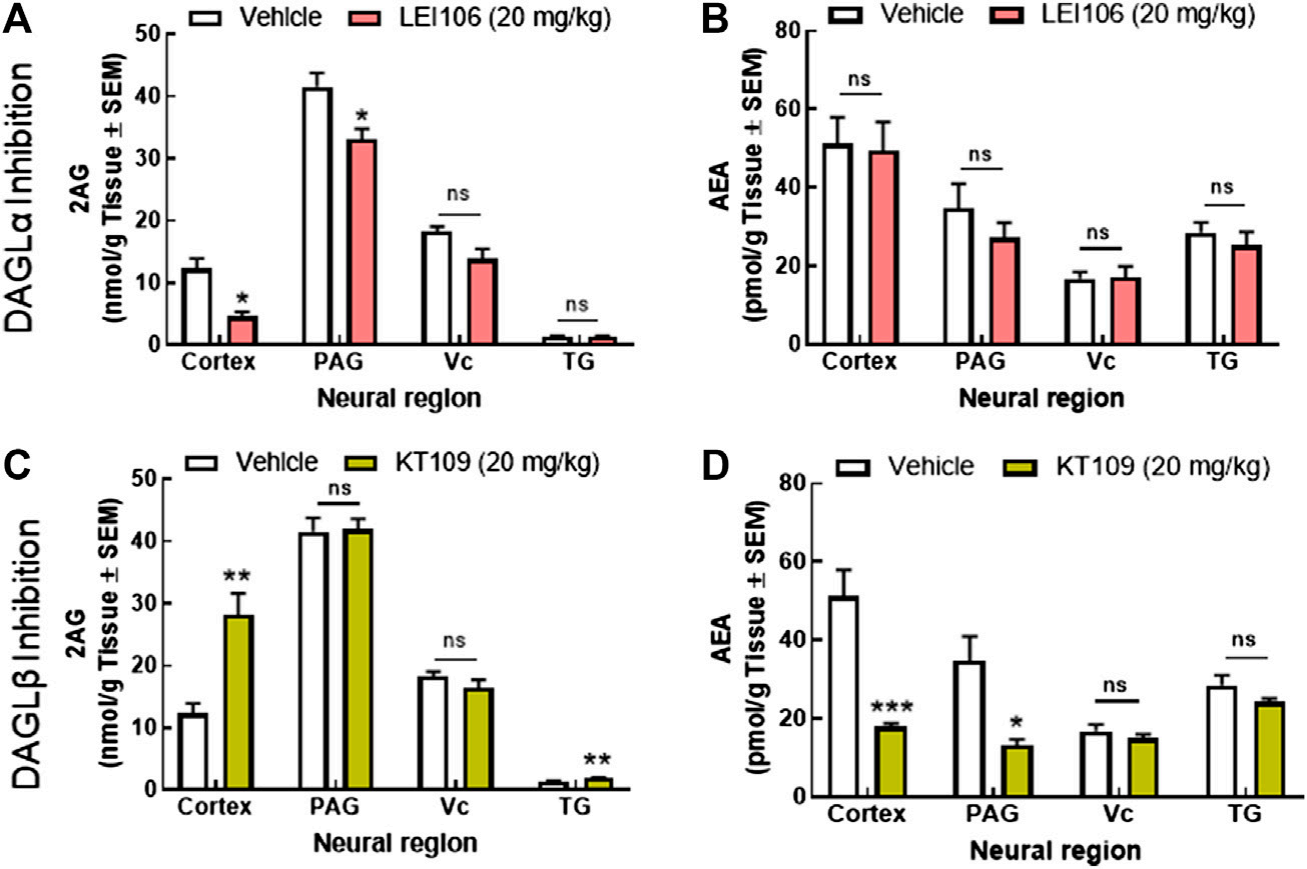
Reduction in cortical and PAG 2-AG, but not AEA is associated with the induction of headache-like pain in female rats. Systemic administration of the DAGLα selective inhibitor, LEI106 (20 mg/kg, IP) significantly reduced 2-AG levels in the cortex and PAG, but not in Vc or TG, as compared to vehicle treated controls **(A)**. There were no significant differences in the level of AEA between vehicle and LEI106-treated rats in any sample evaluated **(B)**. Injection of the DAGLβ selective inhibitor, KT109 (20 mg/kg, IP), significantly increased 2-AG levels in the cortex relative to vehicle treated rats; levels in PAG, Vc, and TG were not changed **(C)**. KT109 significantly reduced AEA levels in both cortex and PAG, but not Vc or TG **(D)**. Data are expressed mean ± SEM (n = 3), one-way ANOVA, *p < 0.05, **p < 0.01, ***p < 0.001, ns, non-significant.

To determine if behavioral outcomes aligned with differences in 2-AG and AEA levels, cortex, PAG, Vc, and TG tissue samples from female rats were collected 120 min after drug administration, and analyzed by LC-MS. Systemic administration of the DAGLα selective inhibitor, LEI106 (20 mg/kg, IP) significantly reduced 2-AG levels in the cortex (V1M) and PAG, but not in Vc or TG, as compared to vehicle treated controls (Figure 9A; LEI106 vs. vehicle Student’s *t*-test by region: Ct: *p* = 0.02, PAG: *p* = 0.03, TG: *p* = 0.68, Vc: *p* = 0.06, n = 3/group). AEA levels were not significantly different between vehicle and LEI106-treated rats in any sample evaluated (Figure 9B; LEI106 vs. vehicle Student’s *t*-test by region: Ct: *p* = 0.87, PAG: *p* = 0.35, TG: *p* = 0.49, Vc: *p* = 0.94, n = 3/group). Dosing with the DAGLβ selective inhibitor, KT109 (20 mg/kg, IP), significantly increased 2-AG levels in the cortex relative to vehicle dosed rats; levels in PAG, Vc, and TG were not changed (Figure 9C; KT109 vs. vehicle Student’s *t*-test by region: Ct: *p* = 0.01, PAG: *p* = 0.91, TG: *p* = 0.01, Vc: *p* = 0.28, n = 3/group). In contrast, KT109 significantly reduced AEA levels in both cortex and PAG, but not Vc or TG (Figure 9D; KT109 vs. vehicle Student’s *t*-test by region: Ct: *p* = 0.008, PAG: *p* = 0.03, TG: *p* = 0.19, Vc: *p* = 0.40, n = 3/group).

To determine if LEI106 induced reductions in 2-AG were coupled to changes in DAG levels, quantitative ELISA was employed. Analysis showed DAG levels (ng/g tissue) in the cortex (Vehicle = 192.17 ± 38.44; LEI106: 145.66 ± 6.29) and PAG (Vehicle = 168.21 ± 7.18; LEI106: 193.21 ± 28.30) that were not significantly different (Student’s *t*-test, cortex *p* = 0.30; PAG *p* = 0.44) (data not shown). Together, these data support loss of 2-AG via inhibition of DAGL in the PAG as a driver of headache pain in this model.

## Discussion

Clinical migraine is associated with sensory sensitivities such as allodynia, photophobia, phonophobia, and hyperosmia (Goadsby et al., 2017). Several animal models have been developed with the aim to understand migraine disorder and to develop new therapeutic strategies (Munro et al., 2017; Harriott et al., 2019; Vuralli, et al., 2019). Although most of these models display similar phenotype to human migraine, there is currently no animal model able to replicate all its features, since migraine is considered a complex disorder with variable phenotypes. Recent clinical studies of migraine sufferers have demonstrated that while episodic migraineurs primarily experience hypersensitivity of solely cephalic sites, chronic migraineurs are more likely to experience both cephalic and extracephalic hypersensitivity (Mathew et al., 2004; Kitaj and Klink, 2005; Bigal and Lipton, 2008; Guy et al., 2010). Similarly, patients with TTH (tension-type headache) solely experience cephalic pain (IHS, 2018). Thus, a reverse translational approach to verifying models for these pain states should focus on the ability of these models to solely induce cephalic hypersensitivity.

Recent reports suggest that functional pain disorders, including migraine/chronic headache, result from deficiencies in the eCB system (Greco et al., 2018a; Greco et al., 2018b; Russo 2016; Tassorelli et al., 2019). Though numerous animal models of migraine-like pain exist (Munro et al., 2017; Harriott et al., 2019; Vuralli, et al., 2019), these models often are invasive or minimally invasive and have not investigated contributions of the ECS to migraine pain. Thus, the studies above investigated the hypothesis that pharmacologic inhibition of DAGL, the enzyme implicated in the synthesis of 2-AG, would induce symptomology aligning with clinical episodic headache. In two established models of medication overuse headache2-AG was reduced in the PAG, while AEA was increased in the cortex. Selective inhibition of DAGLα, but not DAGLβ, induced significant periorbital allodynia in a dose-dependent manner, accompanied with increased secondary behavioral measures of distress (i.e., head pressing) and photophobia, but not reduced movement or anxiety. Behavioral outcomes were associated with significant reductions in 2-AG levels in the cortex and PAG, but not within the trigeminal ganglia or Vc, indicating a CNS origin of headache-like pain; AEA levels were unchanged. Together, these data support 1) DAGLα inhibition as a non-invasive model of episodic headache and 2) identify the 2-AG-ECS system within the PAG as necessary for headache pathology.

The PAG is a point of integration in the descending pain modulatory systems that receives information from cortical and subcortical brain regions and projects to the rostroventromedial medulla (RVM) (Yam et al., 2018). Changes in PAG connectivity have previously been implicated in migraine as well as cannabinoid signaling in this brainstem region (Goadsby et al., 2017). CB_1_R agonists applied in the ventrolateral PAG attenuated dural Aδ trigeminal afferent activation of second order neurons without altering cutaneous activity in the V1 region of the trigeminal nerve; similar effects were observed with the 5HT1B/1D agonist, naratriptan (Akerman et al., 2013). Application of a CB1R antagonist reversed these observations (Akerman et al., 2013). Moreover, AEA did not mediate the same effects in these studies, suggesting that that a second eCB activates these CBRs endogenously. It is also known that 2-AG induces retrograde inhibition (disinhibition) of GABA release via presynaptic CB1 receptors in PAG which leads to antinociception by activating the descending pain inhibitory pathways (Ho et al., 2011; Liao et al., 2011). Interestingly, immunofluorescent staining in these studies demonstrated the presence of DAGLα, but not DAGLβ in PAG. The data collected herein suggest that systemic application of a DAGLα inhibitor selectively depleted 2-AG in the cortex and PAG, whereas DAGLβ increased 2-AG levels in the cortex and reduced AEA levels in the PAG and cortex; in both conditions, eCB levels in the Vc and TG were unchanged. These results implicate 2-AG within the PAG as the endogenous eCB exerting regulation over descending pain modulatory circuits to the trigeminal brainstem complex during headache.

Under normal physiology, 2-AG release is considered on-demand in response to stress, inflammation, and to restore homeostasis (Horne and Stella, 2008). 2-AG acts at CB1R and CB2R to reduce transmitter release with Gαi signaling (Reggio, 2010). Hydrolysis of 2-AG generates arachidonic acid, the precursor substrate for both AEA, and eicosanoid inflammatory signaling (Murataeva et al., 2014). Depletion of 2-AG via DAGLα inhibition with LEI106 is reported to reduce arachidonic acid and eicosanoid levels, elevate DAGs, and impair synaptic plasticity without altering AEA levels *in vitro* and in mice (Janssen et al., 2014; Kohnz and Nomura, 2014); findings above confirm these observations in rats in the cortex and PAG. The pharmacological blockade of DAGLβ by KT109 showed lower levels of 2-AG, arachidonic acid, and prostaglandins in Neuro2A cells, mouse peritoneal macrophages, mouse liver, and human prostate cancer cells (Hsu et al., 2012; Hsu et al., 2013). Activity-based protein profiling confirmed that KT109 is a systemically active DAGLβ inhibitor. DAGLβ in the lumbar spinal cord and brain was blocked after intraperitoneal injection of KT109 at 30 mg/kg dose in rats. However, the treatment with KT109 did not significantly reduce 2-AG, AA, and PGE2 levels in brain, indicating different role of DAGLβ in the central endocannabinoid system (Luk et al., 2018). Our LC-MS results correspond well with those findings. The genetic studies also provided evidence of the different roles of DAGLs in the central nervous system. Dramatic reduction of 2-AG in cortex, cerebellum, hypothalamus, and hippocampus were observed in DAGLα knockout mice, while DAGLβ knockout mice showed lower 2-AG levels only in the hypothalamus, proofing the different contributions of the two isoforms within different brain regions (Gao et al., 2010; Tanimura et al., 2010).

Alternatively, DAGLα inhibition may lead to accumulation of DAG. DAG can activate nonselective cation channels, including TRPV1 and TRPC3, as well as receptor- and store- operated calcium channels to activate nociceptive fibers (Brown and Passmore, 2010; Lafreniere and Kelly, 2018; Yasko et al., 2019). Inhibition of DAGLα did not significantly change DAG levels in the cortex or PAG suggesting that this mechanism may not play a role in the periorbital allodynia resulting from DAGLα inhibition. Rather, periorbital allodynia after blockade of DAGLα may reflect a loss of CB receptor activation on descending pain inhibitory pathways or reduced vascular tone by 2-AG; this is supported by results with sumatriptan and olcegepant. Together, these data point to a central mechanism driving headache pain that is regulated by the 2-AG-eCB system.

Preclinically, medication overuse induced allodynia in animals is observed in both periorbital (central) and hind-paw (peripheral) sites (De Felice et al., 2011). In other acute models, including NO-donor and CSD induction, whole body allodynia is also observed (Fioravanti et al., 2011; Kim et al., 2018). Here, and in contrast to other preclinical models, acute DAGL inhibition induced cephalic hypersensitivity in the absence of hind-paw allodynia, suggesting differences in the role of DAG/DAGL/2-AG signaling in facial vs. somatic regions. Importantly, female rats showed greater sensitivity to DAGLα inhibition as compared to males mirroring sex differences in the clinical presentation of migraine. Relevant to clinical translation, animals with sensitivity after DAGLα inhibitor administration showed positive responses to the clinically used antimigraine agent sumatriptan and the CGRP antagonist, olcegepant.

Non-sensory features of migraine/chronic headache include anxiety, depression, and obesity that increase distress and reduce quality of life (Katsarava et al., 2012). Anxiety disorders are more prevalent in chronic migraineurs (30–50%) vs. episodic (18%) (Katsarava et al., 2012), and depression is reported to precede the transition of episodic migraine to chronic (Ashina et al., 2012). DAGLα inhibition did not reduce voluntary movement or induce anxiety in the open field and elevated plus maze tests, two assays shown to assess behavioral distress (Belovicova et al., 2017), suggesting that acute DAGLα is not necessary for these behaviors. This is notable since these clinical features are indicative of the transition of episodic to chronic migraine (Katsarava et al., 2012). In conclusion, this model recapitulates the pain presentation and behavioral symptomology of episodic, but not chronic, headache.

Limitations: The authors acknowledge that the studies herein have limitations. The drugs applied in these studies might have off-target(s) effects. It is known that LEI106 inhibits the hydrolysis of 2-AG by ABHD6 in mouse brain membrane homogenates and in HEK293T cell membrane preparation (Ki = 0.8 µM) (Janssen et al., 2014). However, the IC50 value of LEI106 (18 nM), targeting sn-1 DAGLα is 40-fold lower, than its off-target ABHD6, proving the higher affinity of LEI106 toward DAGLα. Our LC-MS data showed reduced 2-AG levels in cortex and PAG after the administration of LEI106, aligning with the more potent DAGLα inhibitory effect of LEI106. Notably, KT109 was also reported to inhibit ABHD6 in rodent brain (Hsu et al., 2012; Luk et al., 2018), but our LC-MS results as well as previous report (Luk et al., 2018) did not detect changes in brain 2-AG levels after injection of KT109. KT109 also showed inhibitory activity against PLA2G7 and MAGL at higher concentrations (Kohnz and Nomura, 2014).

Secondly, a genetic approach, targeting Dagla gene would provide additional mechanistic insight into DAG/DAGL/2-AG signaling. Jenniches and co-workers investigated the consequences of the deletion of Dagla gene (Jenniches et al., 2016). 2-AG levels were significantly decreased (by 80–90%) in cortex, hippocampus, striatum, and amygdala in Dagla−/− mice, but reduced AEA levels in cortex and amygdala were also observed. The deletion of Dagla enhanced anxiety, stress, and fear responses, including reduced exploration of the central area of the open field, and increased anxiety-related behaviors in the light/dark box, which were not observed after the acute administration of DAGLα inhibitor, LEI106 in our study. Dagla−/− mice did not show increased pain sensitivity but altered pain response in the hot plate test, drawing the attention to the possible involvement of pain signaling. It is notable that reduced hippocampal neurogenesis was also detected in Dagla−/− mice. The behavioral phenotype of Dagla knock-out animals suggest that the systemic deletion of Dagla affect the emotional state in addition to pain signaling. However, it is not known that this phenotype is due to the acute disruption of 2-AG biosynthesis or rather a consequence of developmental effects. The genetic manipulation of Dagla gene in certain brain area(s) by microinjections would clarify the different roles of DAGLα in pain signaling, anxiety, and stress-responses, but one of the strengths of the present study is the non-invasive feature of pharmacological manipulation of DAGLα would be lost in those experiments.

Our model captured the main features of migraine-like headache, including cutaneous allodynia at the cephalic site, spontaneous pain behavior, like head-pressing and photophobia, however, non-sensory features of migraine such as anxiety, depression were not observed with acute DAGLα inhibition. It is notable that not all the sensory sensitivities associated with clinical migraine, such as phonophobia or hyperosmia were studied in our model system. Several well-validated animal models of headache-like pain exist, each of them has its own advantage and limitation, but nonecan cover all migraine-associated symptomology (Munro et al., 2017; Harriott et al., 2019; Vuralli, et al., 2019; Levine et al., 2020). It is known that 70% of migraineurs experience cephalic allodynia, although extracephalic allodynia in the arms and legs was reported in more severe and chronic cases (Burstein et al., 2000). Anxiety and depression are also more prevalent symptoms in patients with chronic migraine (Ashina et al., 2012; Katsarava et al., 2012). Therefore, symptoms observed after the acute pharmacological blockade of DAGLα more likely represent the episodic stage of migraine-like pain. Whether chronic administration of DAGLα inhibitor could capture signs of chronic form of migraine is an exciting question that needs to be addressed in future experiments.

## Conclusion

Clinical endocannabinoid deficiency is one theory underlying migraine/chronic headache pathology. Drawing on this idea, studies here selectively blocked DAGL to reduce 2-AG which induced symptomology reflective of clinical episodic migraine. DAGLα, rather than DAGLβ, was identified as the critical isoform needed for cephalic pain. Importantly, periorbital sensitivity was reversed by clinical abortive antimigraine agents further supporting the utility of this model to study migraine/chronic headache pathology. Finally, this model of headache via DAGLα inhibition revealed that loss of 2-AG within the PAG is necessary for induction of headache pain. Incorporation of this non-invasive headache model in the field may improve the translation of novel therapeutics and understanding of headache pathology.

## Data Availability Statement

The original contributions presented in the study are included in the article/Supplementary Material, further inquiries can be directed to the corresponding author.

## Ethics Statement

The animal study was reviewed and approved by IACUC approval from the University of Arizona (17-223).

## Author Contributions

AL: Experimental design, data collection/analysis, manuscript preparation. EL-B: Experimental design, data collection/analysis/ and interpretation, manuscript preparation. KK: Data collection and analysis. LG: Data collection and analysis. DS: Experimental design, data interpretation, manuscript preparation. TV: Experimental design, data interpretation, manuscript preparation. TL-M: Experimental design, data analysis/interpretation, manuscript preparation.

## Funding

This work was supported by grants from the National Institute of Neurological Disorders and Stroke (R01NS099292, TL-M) of the National Institutes of Health, Arizona Biomedical Research Commission (ABRC45952, TL-M), with monies from the Department of Pharmacology at the University of Arizona, the M.D.-Ph.D. Program at the University of Arizona, and the University of Arizona Comprehensive Pain and Addiction Center. Research reported in this publication was also supported by the National Cancer Institute of the National Institutes of Health under award number P30 CA023074.

## Disclaimer

Authors are solely responsible for the content which does not necessarily represent the official views of the National Institutes of Health, the State of Arizona, or the University of Arizona.

## Conflict of Interest

The authors declare that the research was conducted in the absence of any commercial or financial relationships that could be construed as a potential conflict of interest.
